# Beyond physical isolation: behavioral correlates and potential neurobiological mechanisms of functional social isolation

**DOI:** 10.3389/fpsyg.2026.1911494

**Published:** 2026-09-29

**Authors:** Xuan Peng, Limin Wang, Dongming Li

**Affiliations:** 1 Hebei Key Laboratory of Animal Physiology, Biochemistry and Molecular Biology, College of Life Sciences, Hebei Normal University, Shijiazhuang, Hebei, China; 2 Hebei Collaborative Innovation Center for Eco-Environment, Hebei Normal University, Shijiazhuang, Hebei, China

**Keywords:** behavioral effects, digital immersion, functional social isolation, intervention, neuroendocrine mechanisms

## Abstract

Functional Social Isolation (FSI) describes a proposed form of social disconnection in the digital era, wherein individuals are physically co-present yet psychologically detached during pervasive digital immersion. This review examines how everyday digital immersion may contribute to persistent social disengagement, outlines its behavioral correlates and potential neurobiological pathways, and discusses its implications for public health and behavioral psychology. By synthesizing emerging literature on co-present digital immersion, social buffering, and neuroendocrine regulation, we identify five behavioral correlates relevant to FSI: attentional fragmentation, habitual or problematic online engagement, emotional volatility, avoidance of offline social interaction, and reduced practice of offline social skills. We also consider evidence concerning oxytocin, dopamine, norepinephrine, endocannabinoids, serotonin, and cortisol, alongside models of internet-related behaviors. These systems may represent candidate pathways linking digital immersion, attentional capture, affective regulation, and reduced engagement with available social partners. However, current neurobiological evidence is largely indirect and is not specific to FSI; similar patterns may arise from traditional social isolation, chronic stress, sleep disturbance, anxiety, or depression. We therefore present a hypothetical multi-system framework intended to generate testable predictions rather than establish an FSI-specific neurochemical state. FSI may be modifiable through interventions that reduce digital interruptions during face-to-face interaction and support meaningful synchronous engagement. Future research should validate its measurement, test causal pathways, and evaluate interventions across populations and social contexts.

## 1 Introduction

Traditional models conceptualize social isolation as an objective physical separation (e.g., solitary housing, maternal separation, incarceration, or enforced group segregation; Vetulani, 2013; Zavaleta et al., 2017). These paradigms consistently link such separation to robust stress-system involvement, including hypothalamic–pituitary–adrenal (HPA) axis dysregulation and an increased risk for mood symptoms (Cacioppo et al., 2011; Nishi, 2020). However, the digital transformation of everyday life has revealed a conceptual gap: a growing form of social detachment that unfolds without physical separation. Individuals may be co-located—in homes, workplaces, classrooms, or public spaces—yet experience diminished mutual attunement and reduced perceived connectedness due to unevenly distributed attention. Consequently, classic isolation models based on spatial separation are insufficient to explain contemporary disconnections that occur in the presence of others.

A key behavioral substrate of this pattern is co-present digital immersion: sustained engagement with highly compelling, algorithmically curated content (e.g., social media feeds, short-form videos, online games) via smartphones or other portable devices, such that attention is repeatedly diverted away from the immediate social environment (Mohamed and Shaban, 2025; Pazdur et al., 2025). Features such as intermittent, unpredictable rewards, personalized cues, and frictionless access can heighten attentional capture and promote habitual checking, potentially displacing face-to-face interaction in contexts that would otherwise support social bonding (Patulny and Seaman, 2017). The scale of exposure is substantial. In the United States, approximately 95% of teenagers report smartphone access, 46% describe being “almost constantly online”, and many report difficulty disengaging from social media (Vogels et al., 2022). Among adults, smartphones are essential for communication and information access (Haug et al., 2015), yet “distraction” and context-inappropriate checking are common motives that interrupt co-present interactions (Levine et al., 2012; Sbarra et al., 2019). As a result, moments that previously supported spontaneous social connections—during commutes, family gatherings, or informal workplace exchanges—may be increasingly fragmented by digital engagement (Walsh and Clark, 2019; Verduyn et al., 2021).

Here, we define functional social isolation (FSI) as a condition in which individuals remain physically present in social environments (e.g., homes, workplaces, or public spaces) yet experience attenuated engagement and reduced interaction quality, reflected in diminished shared attention and weakened subjective connectedness (He and Wang, 2025; Martinsson and Thomée, 2025). FSI overlaps with, but is distinct from, related constructs such as phubbing, technoference, loneliness, social withdrawal, and problematic smartphone use. Phubbing refers to the specific behavior of ignoring a co-present interaction partner because of smartphone use (Arenz and Schnauber-Stockmann, 2024), whereas technoference refers more broadly to technology-related interruptions of interpersonal interaction (McDaniel and Radesky, 2018). Loneliness is a subjective perception of inadequate social connection (Campagne, 2019), and social withdrawal involves reduced participation in or avoidance of social interaction (Li and Wong, 2015). Problematic smartphone use describes excessive or poorly controlled smartphone use across contexts (Horwood and Anglim, 2019). By contrast, FSI refers to the broader state of reduced psychological engagement and interaction quality despite physical co-presence; these related behaviors or experiences may contribute to, or result from, FSI but are not equivalent to it.

This review synthesizes evidence on (1) how co-present digital immersion may contribute to FSI; (2) psychological and behavioral correlates relevant to public health (e.g., loneliness, sleep disruption, and affective symptoms); and (3) plausible neurobiological pathways—particularly stress, reward, and affiliative systems—that may link repeated digital immersion to chronic social stress in everyday life. Given the limited direct neurobiological evidence specific to FSI, these pathways are considered provisional and are informed partly by evidence from related research domains. Together, these perspectives provide a theoretical basis for proposing that FSI may emerge through the interaction of attentional competition, reinforcement-driven digital engagement, and reduced social efficacy. These processes may reinforce one another over time, producing a persistent mismatch between physical social presence and effective psychological engagement.

## 2 Methods

This study employed an integrative literature review approach to synthesize evidence from psychology, behavioral addiction, social neuroscience, and neuroendocrinology and to develop a conceptual framework for FSI. A literature search was conducted using three electronic sources: PubMed, Web of Science, and Google Scholar. Articles published between January 1, 1990, and June 17, 2026, were considered for inclusion.

Keywords included combinations of terms such as “digital immersion,” “smartphone use,” “smartphone addiction,” “problematic smartphone use,” “internet addiction,” “social media addiction,” “digital distraction,” “ocial isolation” “social disconnection,” “social withdrawal,” “social interaction,” “social cognition,” “offline social behavior” and “co-presence.” Additional searches focused on potential neurobiological mechanisms and included “oxytocin,” “dopamine,” “serotonin,” “norepinephrine,” “endocannabinoid,” “cortisol,” “reward processing,” “stress response,” “social reward,” “emotion regulation” and “neuroendocrine regulation.” Boolean operators (AND/OR) were used to combine terms within and across these domains (Carcassi and Sbardolini, 2023).

Publications were considered eligible when they addressed at least one of the following areas: (1) the effects of digital technology use or problematic digital engagement on social behavior or social functioning; (2) behavioral characteristics associated with social disengagement, including attentional disruption, addiction-like engagement, emotional instability, or avoidance of offline interaction; or (3) neurobiological and neuroendocrine mechanisms relevant to social bonding, reward processing, attention, emotion regulation, or stress. Peer-reviewed empirical studies, relevant reviews, and theoretical or mechanistic publications directly relevant to the proposed framework were considered. Publications were excluded if they did not address digital engagement, social functioning, relevant behavioral correlates, or candidate neurobiological mechanisms, or if sufficient information was unavailable to assess their relevance. Reference lists of relevant publications were hand-searched to identify eligible articles. Following full-text assessment, 100 publications were retained for inclusion in this review.

Findings were subsequently organized into behavioral correlates of FSI and potential neuroendocrine mechanisms, with particular attention to interactions among social bonding, reward, attention, emotion regulation, and stress systems. A narrative thematic synthesis was conducted to identify convergent findings, conceptual relationships, and gaps across the included literature.

## 3 Psychological and behavioral correlates of functional social isolation

FSI—being physically near others while experiencing attenuated engagement and reduced interaction quality—pertains to a persistent mismatch between social opportunity and felt connectedness. Unlike overt social withdrawal, FSI is often embedded in routine contexts (family meals, meetings, public spaces) where interaction is possible but repeatedly interrupted by digital immersion (Walsh and Clark, 2019). Its central feature is a split between presence and connection: individuals may share a space, yet their core needs for empathy, trust, and mutual understanding may remain unmet (Bertolín-Guillén, 2023). This chronic gap between the potential for and the reality of connection may function as a source of recurring social stress without requiring physical isolation or deliberate avoidance. It may be expressed as reduced engagement with the immediate social environment, accompanied by a sense of isolation even in the presence of others (Bayram and Yilmaz, 2024). The impact of this isolation may be cumulative. Over time, these micro-disruptions may accumulate into lower perceived empathy, reduced trust, and diminished relationship satisfaction, thereby contributing to five interrelated psychological and behavioral correlates: (1) attentional fragmentation, (2) compulsive or habitual checking, (3) affective volatility, (4) avoidance of high-cost offline interaction, and (5) potential skill erosion through reduced practice of face-to-face social cue processing (Figure 1).

**Figure 1:**
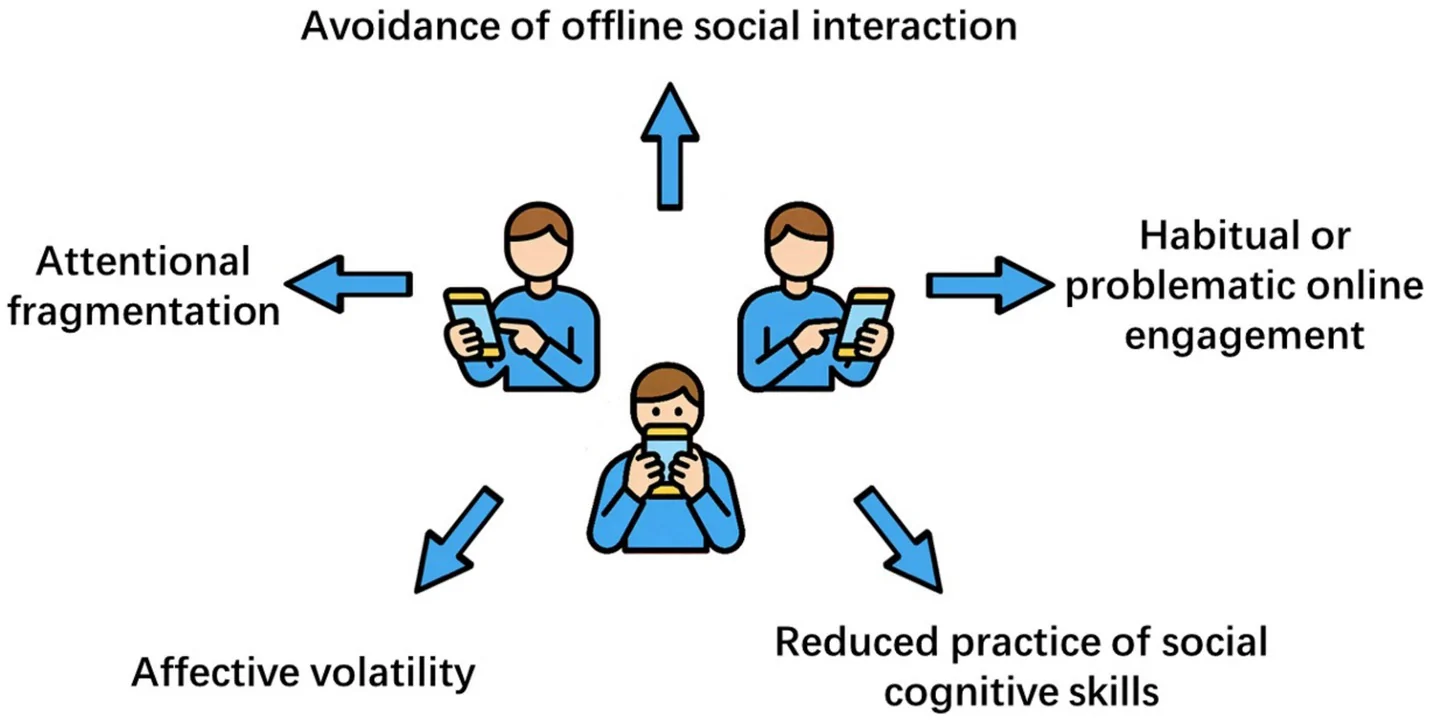
Behavioral correlates of functional social isolation (FSI). Schematic illustration of individuals who are physically present in a social setting but psychologically disengaged during pervasive digital immersion. The five behavioral correlates shown are: (1) attentional fragmentation; (2) habitual or problematic online engagement; (3) affective volatility; (4) avoidance of offline social interaction; and (5) reduced practice of social cognitive skills. Together, these domains reflect the proposed defining feature of FSI: diminished shared attention and perceived connectedness despite close physical proximity.

### 3.1 Attentional fragmentation

In FSI, the most proximal behavioral change is attentional fragmentation during co-present social situations. Contemporary digital platforms draw attention through salient cues (e.g., notifications, badges, autoplay, and infinite scrolling). In face-to-face interaction, maintaining conversation quality depends on sustained attention to social signals (eye gaze, facial affect, prosody, turn-taking) and the capacity to maintain a shared conversational thread. When digital cues repeatedly compete for attentional resources, interruptions may reduce the continuity of this shared attentional state and weaken the reciprocal processing of eye gaze, facial affect, prosody, and conversational feedback. Thus, attentional fragmentation is theoretically positioned as the most proximal process through which digital immersion may reduce social efficacy despite continued physical co-presence.

The social impact is not limited to overt phone use. Field and laboratory studies suggest that merely having a mobile device present can reduce perceived closeness, empathic connection, and conversation quality, consistent with devices acting as “attention affordances” and competing goals in co-present contexts (Misra et al., 2016). Mechanistically, FSI may involve more than transient distraction; repeated attentional capture and switching may disrupt joint attention—the shared allocation of attention that supports mutual understanding and emotional attunement. Rapid, successive judgments about fragmented stimuli (e.g., like, swipe, click) may increase cognitive load and leave fewer attentional resources available for processing complex social information (Oyasor, 2025). Such demands may recruit executive-control processes supported by the prefrontal cortex (PFC), although direct neural evidence specific to FSI remains limited. Repeated attentional switching may therefore make sustained, high-quality face-to-face engagement more difficult, rather than necessarily producing a generalized or permanent change in the brain.

### 3.2 Habitual and problematic online engagement

A key conceptual refinement is to distinguish habitual checking from problematic smartphone use (PSU) rather than treating all high-frequency use as addiction. Habitual checking refers to cue-triggered, automatic engagement (e.g., unlocking the phone during micro-pauses), often driven by learned associations between contextual cues (silence, boredom, notification sounds) and brief rewards (Roffarello and De Russis, 2021). Habitual checking can occur without major functional impairment, yet can still contribute to FSI by interrupting co-present interaction at high frequency. PSU describes a clinically relevant pattern marked by loss of control and functional impairment (e.g., sleep interference, work/study disruption, or relationship strain), often with craving-like urges and unsuccessful attempts to cut back (Jahrami et al., 2022; Parasuraman, 2023; Srivastava et al., 2025). This distinction matters for public health framing: FSI can arise from widespread habitual interruption in everyday settings, while PSU represents a higher-severity subgroup with stronger links to mental health symptoms and life impairment.

Theoretical models can help organize these pathways. The interaction of person-affect-cognition-execution (I-PACE) model explains how vulnerabilities (e.g., trait impulsivity, social anxiety), affective states (stress, boredom), cognitive biases (expectancies, attentional bias), and executive control interact with platform features to promote escalating use and dysregulation (Brand et al., 2016). Compensatory/negative reinforcement accounts propose that some individuals use online engagement to cope with distress or unmet offline needs, strengthening reliance on the device and amplifying avoidance of offline interaction (Kardefelt-Winther, 2014). Framing FSI within these models yields clearer hypotheses: habitual checking may be most sensitive to cue control (e.g., notification management), whereas PSU may require broader interventions targeting coping motives, sleep, and self-regulation.

Immersive digital interaction often involves uncertain or variable rewards: users cannot predict whether the next refresh will reveal social salient, entertaining, or personally relevant content. Evidence from reward-learning research suggests that reward uncertainty can engage anticipatory and reinforcement-related processes involving the ventral striatum (Tanaka et al., 2006). By extension, such processes may help explain repeated checking and refreshing, although evidence from general reward paradigms should not be interpreted as direct evidence of smartphone-related neural effects. Repeated engagement may gradually become habitual and increasingly driven by contextual cues rather than the value of particular content (Meinhardt et al., 2025). Attempts to resist checking may also be accompanied by discomfort or anxiety, particularly among individuals with problematic patterns of use (Elhai et al., 2016). The consequence may be reduced autonomous control over attention, crowding out time and attentional resources that could otherwise be devoted to face-to-face interaction. Within the present framework, these processes provide a theoretical explanation for how repeated digital engagement can shift attentional and behavioral resources away from available offline social interactions.

### 3.3 Affective volatility

FSI may be associated with affective volatility, though mechanistic claims should be interpreted cautiously. Research has shown that social media use is associated with increased technostress and exposure to cyber incivility (Mert et al., 2025). Three converging processes may be involved: (1) affective micro-fluctuations associated with intermittent and unpredictable rewards; (2) social comparison and emotionally arousing content that can heighten negative affect in susceptible individuals (Kross et al., 2013; Aalbers et al., 2019); and (3) sleep disruption associated with evening or night-time device use, which may impair emotion regulation and increase next-day reactivity. Experimental and epidemiologic studies link certain patterns of passive digital consumption to poorer wellbeing, particularly among adolescents and young adults (Roberts and David, 2023). In this framing, affective volatility may be amplified by sleep-related vulnerability and may reduce the capacity for steady offline emotional engagement.

Notifications, social feedback, and personally relevant content can produce brief positive affect and anticipatory engagement (Sherman et al., 2018). Because these rewards are often intermittent and short-lived, users may repeatedly seek novel or salient content, particularly when experiencing boredom, stress, or negative affect (Wang and Lee, 2020). Interruption of ongoing digital engagement may consequently elicit frustration or irritability in some individuals, especially when use has become habitual or difficult to control. Frequent switching between highly stimulating digital content and lower-stimulation offline settings may also be associated with boredom, restlessness, and difficulties sustaining attention (Marty-Dugas et al., 2018; Dunne, 2025). These patterns may undermine stable emotion regulation and reduce engagement with the slower and less immediatedly reinforcing emotional exchanges characteristic of many face-to-face interactions. However, direct evidence that these effects are mediated by dopamine or constitute tolerance and withdrawal remains limited.

### 3.4 Avoidance of real-world social interaction

FSI should not be framed solely as passive disconnection. Real-world social interaction is complex, relatively uncontrollable, and associated with the risk of rejection or misunderstanding. Studies suggest that fear of missing out (FoMO) is positively associated with problematic smartphone use, which may undermine friendship satisfaction. Phubbing can also reduce friendship satisfaction, although reciprocal phubbing may alter this association (Tufan et al., 2025). For some individuals, shifting resources toward digital interaction may represent a strategic means of reducing the perceived costs of offline interaction (Hutchins et al., 2021). Digital interfaces offer comparatively high control over engagement by allowing users to edit messages, curate self-presentation, and disengage easily. Rewards on these platforms, including likes and comments, are quantifiable and immediate (Son and Oh, 2023), potentially making online engagement more attractive when offline interaction is perceived as high-cost or uncertain. Selection effects and reverse causality are important: loneliness or distress can predispose individuals to retreat into digitally mediated interaction, which may then displace offline engagement and maintain distress. Compensatory internet use models predict this bidirectionality, not a one-way causal chain (Kardefelt-Winther, 2014; Brand et al., 2016). Such avoidance may be adaptive in the short-term, but persistent reliance on it may reduce opportunities to develop confidence, reciprocity, and coping skills in face-to-face interaction (Kross et al., 2013; Aalbers et al., 2019). Under these conditions, digital mediated relationships may not always provide the same forms of embodied, contextual, and reciprocal support as sustained offline interaction.

### 3.5 Reduced practice of social cognitive skills

Social cognitive skills are supported and refined through repeated face-to-face interaction. Reduced opportunities to interpret facial expressions, vocal nuances, body language, and conversational timing may therefore limit practice of these skills, although evidence for generalized or permanent impairment remains limited (Wajahat, 2024). Possible manifestations include greater difficulty interpreting ambiguous social cues, weaker perceived empathy, and discomfort during group interaction (Sbarra et al., 2019). These difficulties may create bidirectional social friction: individuals may become frustrated or less confident, while others may interpret reduced responsiveness as disinterest, potentially reducing subsequent opportunities for interaction. Consequently, the perceived threshold for re-engaging in face-to-face interaction may rise, reinforcing FSI. However, the resulting changes may reflect reduced use of particular social skills rather than deterioration of social capacity. This interpretation suggests that any reduction in offline social efficacy may be experience-dependent and potentially reversible (Silva, 2025). Social cognition is supported and refined through repeated exposure to reciprocal interaction, including eye contact, conversational timing, emotion recognition, and the interpretation of nonverbal feedback. If repeated digital engagement displaces opportunities to practice these processes, the resulting changes may reflect reduced use of particular social skills rather than a generalized loss of social capacity. It suggests that the proposed erosion of offline social skills is potentially reversible and dependent on patterns of social experience.

Reduced practice may also influence the efficiency with which face-to-face social cues are processed. Evidence indicates improved recognition of nonverbal cues after a period of reduced screen-based media access (Uhls et al., 2014). We propose that repeated digital immersion may reduce opportunities for sustained eye contact, turn-taking, and affective attunement, potentially lowering performance on tasks requiring rapid integration of facial and vocal cues and diminishing perceived connectedness during conversation. This hypothesis is testable using observable indicators, including: (1) eye contact and gaze sharing (e.g., proportion of time attending to the interaction partner’s face and gaze synchrony); (2) turn-taking dynamics (e.g., response latency, interruptions, conversational repair after interruptions); (3) emotion-recognition tasks (e.g., facial-affect identification, prosody recognition, and sarcasm or irony detection); and (4) post-interaction outcomes (e.g., perceived closeness, empathy, relationship satisfaction).

The five behavioral correlates discussed above can be viewed as interconnected expressions of the same underlying process rather than as independent consequences of digital use. Attentional fragmentation represents the immediate disruption of social information processing; repeated reinforcement may increase habitual or addiction-like digital engagement; affective volatility can increase reliance on rapid digital stimulation for short-term regulation; avoidance may reduce exposure to the uncertainty and demands of offline interaction; and reduced practice may contribute to lower social-cognitive performance ore efficacy. In this way, the proposed FSI framework links moment-to-moment attentional competition with longer-term changes in behavioral preference and social functioning.

## 4 Neuroendocrine effects of functional social isolation

This section synthesizes plausible neuroendocrine mechanisms that may link co-present digital immersion to chronic social stress in real-world settings (Figure 2, Table 1). Importantly, the evidence reviewed should not be interpreted as demonstrating a direct or exclusive causal pathway from digital immersion to specific neuroendocrine alterations. Similar neuroendocrine changes can also arise from other environmental and psychological conditions, including physical or perceived social isolation, chronic stress, sleep disruption, anxiety, depression, and other forms of behavioral addiction. We emphasize that direct causal evidence in humans is still emerging; therefore, the proposed relationship between digital immersion and these neurobiological systems is best understood as a context-dependent and potentially interacting process rather than a simple one-directional causal pathway. On this basis, the neuroendocrine systems discussed below are considered as potential mechanisms that may be relevant to the behavioral processes associated with FSI, rather than isolated biological causes of FSI. Much of the mechanistic account is derived from related research on conventional social isolation, stress physiology, reward learning, and problematic digital use. Accordingly, the framework below presents testable hypotheses rather than established FSI-specific mechanisms. The brain is highly susceptible to environmental influence, and the neural networks governing social behavior and emotional regulation are shaped by the social milieu (Boyce, 2016). Chronic social isolation can exert profound effects on the brain, including endocrine changes that accompany behavioral and psychological alterations (Cruces et al., 2014; Pietrabissa and Simpson, 2020). In humans and non-human mammals, these processes are relevant to the development and progression of social stress and related outcomes (Mumtaz et al., 2018; Ohline and Abraham, 2019).

**Figure 2:**
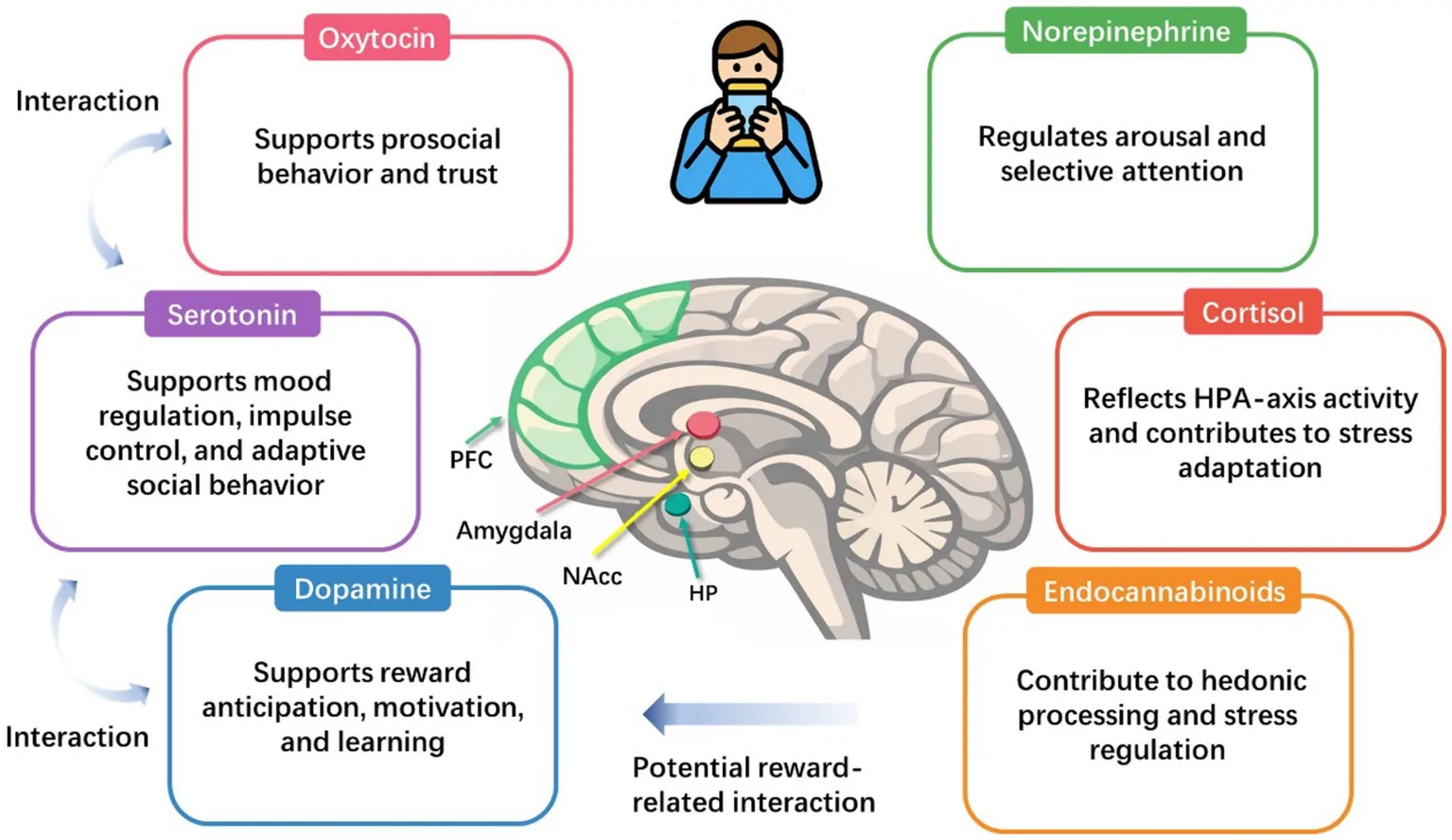
Proposed multi-system neurobiological framework relevant to functional social isolation (FSI). The schematic presents candidate neurobiological systems that may be relevant to the association between co-present digital immersion and the behavioral correlates of FSI. The central diagram highlights brain regions involved in social, affective, attentional, and reward processing, including the amygdala, nucleus accumbens (NAcc), hippocampus (HP), and prefrontal cortex (PFC). The surrounding boxes summarize the broader functions of oxytocin (OT), norepinephrine (NE), cortisol, endocannabinoids (EC), dopamine (DA), and serotonin (5-HT). These systems may contribute to variations in social engagement, attention, reward learning, mood regulation, and stress responses. However, evidence for their involvement in FSI is currently limited and largely indirect, drawing partly on research concerning conventional social isolation, chronic stress, problematic digital use, reward-related behavior, and animal models. The arrows represent hypothesized cross-system interactions rather than established causal pathways. Accordingly, this framework is intended to generate testable hypotheses and should not be interpreted as evidence of an FSI-specific neurobiological state.

**Table 1:** Representative evidence on neuroendocrine systems relevant to social behavior, attention, reward, emotion regulation, and stress across human and rodent models.

Behavior	Hormone	Species	Neuroendocrine change	Behavioral change	Reference
Inattention	Norepinephrine	Human	Extracellular NE level increase	Random exploration decreases	Warren et al., 2017
		Mouse	LC-PrL neural activity recruit	Sustained attention increases	Hallock et al., 2024
		Mouse	LC neurons activity reduces	Hyperlocomotion and attention deficits	Brannan et al., 2024
Addiction	Dopamine	Human	Dopamine release in reward region	Social media addiction	Thangavel, 2024
		Mouse	VTA-NAcore pathway active	Cocaine-seeking behavior triggered	Jing et al., 2022
		Rat	Dopamine release reduces	Fentanyl-CPP development reduced	Sustkova-Fiserova et al., 2020
	Endocannabinoid	Human	AEA and 2-AG concentrations increase	Gambling disorder	Baenas et al., 2023
		Mouse	CB1R activity is inhibited	Anxiety-like behavior and reduce ability of reward learning	Cui et al., 2025
Emotion	Serotonin	Human	Tryptophan level decrease	Annoyance potentiated	Kanen et al., 2021
		Mouse	5-HT4R gene knockout	Anxiogenic phenotype	Karayol et al., 2021
		Mouse	Exposure to 5-HT antagonist in prenatal period	Anxiety-like behavior	Yu et al., 2019
Prosociality	Oxytocin	Human	Intranasal oxytocin provided	Cooperation probability increase	Cosme et al., 2025
		Human	Oxytocin level increase	Trusting behavior increase	Vogt et al., 2025
		Mouse	Oxytocin level increase	Impaired social behavior restored	Takemoto et al., 2022

Evidence summarized in this table provides indirect mechanistic background for the proposed FSI framework and should not be interpreted as direct empirical evidence of neuroendocrine alterations caused by FSI.

### 4.1 The oxytocin system—the social affiliative interface

Oxytocin (OT) coordinates social motivation, empathy, emotion regulation, and stress resilience via widespread projections to social and limbic regions (amygdala, nucleus accumbens (NAcc), hippocampus, and PFC; Dölen and Malenka, 2014; Cheng et al., 2016). OT enhances social cue salience, fosters trust, and interacts with dopaminergic reward circuits to augment the perceived value of social engagement (Baskerville and Douglas, 2010; Olff et al., 2013). In addition, OT contributes to stress buffering by modulating HPA-axis activity and amygdala reactivity to social threat, thereby supporting flexible and prosocial responses (Yeğen, 2010).

In the context of FSI, prolonged co-presence accompanied by high-frequency digital stimulation may reduce opportunities for high-quality face-to-face exchange and may therefore alter the social contexts in which endogenous OT-related processes are normally engaged. However, changes in OT activity may also occur in response to physical social isolation, chronic psychosocial stress, disrupted attachment, anxiety, and other conditions involving reduced or impaired social interaction. We propose that persistent digital distraction may reduce opportunities for the high-quality social interactions that normally engage OT-related affiliative and stress-buffering processes. It is possible that such changes in social experience are associated with differences in phasic OT activity or receptor-level responses, but these possibilities have not been directly established in FSI (Braga-Dias et al., 2025; Papasteri et al., 2020). Potential consequences may include reduced social salience, diminished affiliative motivation, and less effective processing of social signals, such as eye gaze, facial expressions, and prosody, which may weaken the perceived reward value of offline interaction (Hazani et al., 2025; Yao and Kendrick, 2025). Because OT is involved in the prefrontal-limbic networks relevant to empathy, stress regulation, and social attention, altered OT-related functioning may provide one candidate pathway linking reduced affiliative engagement to lower perceived connectedness (Koch et al., 2016; Zhao et al., 2017). OT-related processes may also interact with functional connectivity within broader social-brain networks involved in social memory and stress regulation (Alaerts et al., 2019; Menon and Neumann, 2023). Importantly, OT effects are context-dependent and may vary according to individual history, sex, and current affective state (Ma et al., 2018). Empirical testing should combine multimodal biomarkers, task-based social paradigms, and neuroimaging, ideally within longitudinal designs that capture within-person variability and individual differences in OT-related responses.

### 4.2 Dopamine system—reward anticipation, motivation, and habit formation

The dopaminergic system drives reward anticipation, motivation, and habit formation. The mesolimbic pathway, originating in the ventral tegmental area (VTA) and projecting to the NAcc, underpins reward expectation, goal-directed motivation, and reinforcement learning, and is especially sensitive to novel, unpredictable, and immediate rewards (Dayan and Balleine, 2002; Macit et al., 2018). Digital environments provide salient and often intermittent rewards, including likes, novel content, and brief social cues, which may engage dopamine (DA)-related reward-learning processes and strengthen repeated patterns of use (Macit et al., 2018; Thangavel, 2024). However, direct evidence that everyday digital content reliably triggers specific patterns of phasic DA release remains limited. Repeated exposure to rapidly delivered digital rewards may increase their motivational salience relative to slower or less predictable offline rewards, potentially reducing engagement with available face-to-face interaction (Volkow et al., 2010). The proposed relevance of DA to FSI lies in the possibility that rapid, intermittent, and personalized digital rewards repeatedly recruit general reward-learning mechanisms in situations in which offline social interaction is simultaneously available but behaviorally deprioritized.

Repeated exposure to digital reward cues may bias attention and behavior toward online stimuli at the expense of offline social engagement. DA-related reward and executive-control processes may be relevant to sustained attention, motivation selection, and emotional responses to salient stimuli (Ott and Nieder, 2019; Wang et al., 2020). However, evidence from general reward paradigms should not be taken to demonstrated that digital engagement produces repeated “dopaminergic surges” or directly impairs prefrontal regulation may nevertheless make sustained engagement with slower-paced social interaction more difficult (Honma et al., 2022). Thus, FSI may involve a context-dependent shift in the relative salience of digital and offline rewards, rather than a generalized reduction in reward capacity or a permanent “over-tuning” of the DA system.

### 4.3 Serotonin system—impulse control and emotional regulation

The serotonin (5-HT) system, originating primarily from the dorsal raphe nucleus (DRN) and projecting to PFC, amygdala, and other limbic regions, plays a central role in mood regulation, impulse control, and adaptive social behavior. It supports emotional stability, moderates aggression, and contributes to flexible social decision-making (Runions et al., 2019; Tian et al., 2017). Dysfunctional 5-HT signaling is linked to emotional lability and impulsivity, increasing vulnerability to boredom, anxiety, and irritability, which can drive more frequent, rapid use of digital devices to regulate emotions (Kranz et al., 2010). Conversely, interruptions to immersive digital engagement may be accompanied by irritability or distress in individuals with habitual or problematic patterns of use. Although serotonergic processes may be relevant to impulse control and emotion regulation, direct evidence that reduced serotonergic tone causes withdrawal-like responses to interrupted digital use is currently insufficient (Belmer et al., 2015).

In FSI, chronic digital immersion involving rapid reward cycles and social comparison may interact with serotonergic processes relevant to negative-affect regulation and impulse control. This could shift behavioral preference toward immediate digital relief when real-world interaction is perceived as demanding or uncertain, contributing to irritability and reduced patience in offline interactions. Importantly, 5-HT interacts with other systems involved in social motivation and reward, including OT- and DA-related processes: 5-HT modulates social cue processing and reward salience, and concurrent alterations across these systems may contribute to social disengagement (Dölen, 2015; Fischer and Ullsperger, 2017). Moreover, the influence of 5-HT on emotional regulation and social behavior is context-dependent, with receptor-subtype- and circuit-specific effects that may vary across individuals and situations (Duerler et al., 2022). Although chronic exposure to highly stimulating digital environments has been proposed to affect processes associated with serotonergic regulation, direct evidence of disrupted 5-HT homeostasis in FSI is not yet available (Chandavat et al., 2025). Pre-existing differences in serotonergic function, mood regulation, or anxiety may increase vulnerability to excessive digital engagement, while persistent digital-use patterns may, in turn, contribute to emotional dysregulation indirectly through sleep disruption, repeated reward cycles, or reduced engagement in offline activities.

### 4.4 Norepinephrine system—arousal, attention, and the attention gatekeeper

The locus coeruleus–norepinephrine (LC-NE) system coordinates arousal and selective attention, enabling rapid responses to salient stimuli. Originating in the locus coeruleus and projecting broadly to cortical and subcortical regions, NE modulates sensory gain and cognitive control by shifting the balance between tonic arousal and phasic responses to task-relevant cues (Schwarz and Luo, 2015). Phasic NE bursts enhance the processing of relevant events, while tonic NE levels set the baseline for vigilance and readiness to respond to environmental demands (Gabay et al., 2011).

Repeated exposure to rapidly changing and salient digital stimuli interacts with LC-NE processes involved in arousal and attentional selection (Hellriegel and D'mello, 1997). In co-present settings, high-salience digital cues may compete with subtler and slower-paced social cues for limited attentional resources, potentially making sustained face-to-face engagement feel less immediately stimulating (Berridge, 2008). However, direct evidence that digital immersion chronically overactivates or adaptively reshapes the human LC–NE system remains limited. Within the proposed FSI framework, the LC-NE system may therefore be viewed as a candidate mechanism through which differences in stimulus salience and arousal influence attentional allocation. Repeated competition between rapidly changing digital stimuli and slower-paced offline engagement may bias attention toward the former, particularly among individuals with elevated arousal needs or reduced attentional control. This proposed pathway should be tested using physiological and neuroimaging measures rather than interpreted as an established neurobiological feature of FSI.

### 4.5 Endocannabinoid system—immediate hedonic relief and habit formation

The endocannabinoid (EC) system modulates hedonic experience and stress resilience and interacts with dopaminergic reward circuits to support habit formation (Wenzel and Cheer, 2018). It comprises endogenous cannabinoids (notably anandamide and 2-AG), their synthesizing and degrading enzymes (e.g., FAAH, MAGL), and cannabinoid receptors (primarily CB1 and CB2). EC signaling is involved in hedonic motivation, stress regulation, and reward-related processes through endogenous ligands such as anandamide and 2-AG (Zona et al., 2017).

Engagement with attractive digital content can provide rapid experiences of pleasure or relief, particularly under conditions of boredom or stress. The EC system may be relevant to these experiences because of its established roles in hedonic processing, stress regulation, and interactions with dopaminergic reward pathways (Zona et al., 2017). However, direct evidence that digital content activates EC signaling, or that FSI involves chronic EC-system alterations, is currently limited. More broadly, interactions between EC and dopaminergic systems may support reinforcement learning and habit formation (Covey et al., 2017), providing a possible mechanism through which repeated device use becomes increasingly cue-driven and automatic. Within the FSI framework, this process may contribute to a behavioral preference for immediately available digital gratification over more effortful or uncertain offline interaction, but it should be regarded as a testable hypothesis rather than an established pathway.

### 4.6 Cortisol—chronic low-level stress and HPA-axis dynamics

Cortisol, an end-product of HPA-axis activity, is involved in physiological responses to stress and adaptation (Saxbe, 2008). Chronic psychosocial stress can alter diurnal cortisol rhythms and affect cognitive and emotional regulation (Russell and Lightman, 2019). Within the proposed FSI framework, a persistent mismatch between available social contact and experienced connectedness may function as a recurring psychosocial stressor. Device-related arousal, sleep disruption, and unmet social needs may also interact with HPA-axis regulation (Russell and Lightman, 2019; Thomson, 2019). However, direct evidence that FSI produces sustained HPA-axis activation or elevated basal cortisol is currently lacking. Accordingly, cortisol is considered here as a candidate marker of stress-related processes that may accompany FSI, rather than an established endocrine consequence of it. Longitudinal studies using repeated cortisol measurements are needed to determine whether FSI is associated with altered basal levels, diurnal rhythms, or stress reactivity.

Elevated or dysregulated cortisol activity under chronic stress can influence PFC-dependent inhibitory control, attention, and decision-making, particularly during development (Ma et al., 2017; Verdejo-Garcia et al., 2015). Stress-related reduction in self-regulatory capacity may make it more difficult for some individuals to limit habitual device use or resist immediately rewarding digital stimuli. Cortisol-related stress responses may also interact with amygdala reactivity, anxiety, and negative affect (van Stegeren et al., 2007). Under such conditions, digital engagement may serve as a short-term emotion-regulation strategy by providing distraction or rapidly available reward. This pattern may, in turn, reduce opportunities for offline social engagement. Thus, HPA-axis processes offer a plausible—but currently indirect—perspective on how recurring social stress, impaired self-regulation, and digital coping may interact in FSI. These relationships are likely to be bidirectional and influenced by sleep, pre-existing anxiety or depression, developmental stage, and the broader social environment.

### 4.7 Multi-system interactions

Based on the behavioral and neurobiological evidence reviewed here, we propose a hypothetical framework linking FSI to the interaction of multiple neuroendocrine systems. This framework does not assume a single, linear pathway in which digital immersion directly produces a defined neurochemical state and subsequently causes FSI. Instead, the neurobiological processes discussed above are shared across multiple forms of social stress, behavioral reinforcement, emotional dysregulation, and environmental stimulation. FSI may emerge when general processes involved in reward, arousal, social bonding, emotion regulation, and stress are repeatedly engaged in the distinctive context of physical co-presence accompanied by reduced effective social engagement.

Within this framework, DA-, NE-, and EC-related processes may contribute to reward learning, arousal, and the development of habitual digital engagement, whereas OT-related processes may be relevant to affiliative motivation and social stress buffering. The HPA-axis and cortisol may reflect or modulate stress associated with persistent discrepancies between available and experienced social connection, while 5-HT-related processes may influence mood, impulse control, and tolerance of social uncertainty. These systems are not independent, and their interactions may vary according to developmental stage, sex, prior social experience, mental health, sleep, and patterns of digital use.

The proposed cross-system relationships should be interpreted as hypotheses derived from converging but indirect evidence. For example, stress-related processes may interact with affiliative signaling, and reward-learning processes may influence whether attention is allocated to digital or co-present social stimuli. However, the directions, magnitude, and temporal sequence of these relationships have not been established specifically for FSI. Longitudinal and experimental studies combining behavioral assessments with neuroendocrine and neuroimaging measures are required to test whether these systems predict the emergence, persistence, or resolution of FSI. Overall, the framework illustrates how general mechanisms of reward, arousal, stress, emotion regulation, and social bonding may converge in the context of persistent digital engagement during physical co-presence.

## 5 Conclusions and future directions

FSI describes a proposed form of social disconnection in which individuals remain physically present with others, but experience reduced engagement, interaction quality, and subjective connectedness. Co-present digital immersion may contribute to this mismatch by repeatedly diverting attention from face-to-face interaction. However, digital technology does not inevitably impair social connection; its effects depend on patterns of use, individual vulnerability, and social context. This review identifies five interrelated behavioral correlates of FSI: attention fragmentation, habitual or addiction-like digital engagement, affective volatility, avoidance of demanding offline interaction, and reduced practice of social cognitive skills. These processes may reinforce one another over time, although their directionality remains uncertain. Pre-existing loneliness, anxiety, sleep disruption, or social difficulties may increase reliance on digital interaction, while repeated digital interruption may maintain or intensify these problems.

OT-, DA-, NE-, 5-HT, EC-, and cortisol-related processes may be relevant to the affiliative, reward, attentional, emotional, and stress mechanisms associated with FSI. Nevertheless, these systems should not be interpreted as isolated causes of FSI or as evidence of an FSI-specific neurochemical state. Much of the available evidence is indirect and derived from research on conventional social isolation, stress, reward learning, and problematic digital use. The proposed multi-system framework should therefore be regarded as a set of testable hypotheses rather than an established causal model. Interventions should focus not simply on reducing overall screen time, but on limiting digital interruptions when face-to-face interaction is available. Potential strategies include device-free periods during meals, meetings, and social activities; reduced nonessential notifications; platform designs that facilitate intentional disengagement; and programs that strengthen attentional control, emotion regulation, and offline social confidence.

Future research should first establish whether FSI is empirically distinct from phubbing, technoference, loneliness, social withdrawal, and problematic smartphone use. A validated measure is needed to assess the mismatch between physical co-presence and psychological engagement, ideally combining self-reports with device-use data, gaze behavior, conversational turn-taking, and perceived interaction quality. Longitudinal and experimental studies are also required to clarify directionality and causality. Researchers could manipulate device presence or notification availability during social interaction and assess changes in shared attention, perceived closeness, and affect. Studies combining behavioral measures with validated physiological or neural indicators may help test the proposed mechanisms, while accounting for sleep, mental health, developmental stage, sex, and social context. Cross-cultural and population-specific studies should further examine how FSI varies across families, classrooms, workplaces, and age groups.

FSI remains a proposed conceptual framework rather than an established clinical construct, and no validated FSI-specific measure is currently available. The reviewed literature is heterogeneous, and much of it is cross-sectional or correlational, limiting causal conclusions. Moreover, the proposed neuroendocrine pathways are based largely on indirect evidence from adjacent research domains and should not be interpreted as mechanisms specific to FSI. Finally, because this was an integrative rather than a systematic review or meta-analysis, selection and publication biases cannot be excluded. The framework should therefore serve primarily as a basis for future hypothesis-driven research.

## References

[ref1] Aalbers G. McNally R. J. Heeren A. De Wit S. Fried E. I. (2019). Social media and depression symptoms: a network perspective. J. Exp. Psychol. Gen. 148, 1454–1462. doi: 10.1037/xge0000528, 30507215

[ref2] Alaerts K. Bernaerts S. Vanaudenaerde B. Daniels N. Wenderoth N. (2019). Amygdala–hippocampal connectivity is associated with endogenous levels of oxytocin and can be altered by exogenously administered oxytocin in adults with autism. Bio. Psychiat. Cogn. N. 4, 655–663. doi: 10.1016/j.bpsc.2019.01.008, 30846366

[ref3] Arenz A. Schnauber-Stockmann A. (2024). Who “phubs”? A systematic meta-analytic review of phubbing predictors. Mob. Media Commun. 12, 637–661. doi: 10.1177/20501579231215678

[ref4] Baenas I. Solé-Morata N. Granero R. Fernández-Aranda F. Pujadas M. Mora-Maltas B. . (2023). Anandamide and 2-arachidonoylglycerol baseline plasma concentrations and their clinical correlate in gambling disorder. Eur. Psychiatry 66:e97. doi: 10.1192/j.eurpsy.2023.2460, 37937379 PMC10755577

[ref5] Baskerville T. A. Douglas A. J. (2010). Dopamine and oxytocin interactions underlying behaviors: potential contributions to behavioral disorders. CNS Neurosci. Ther. 16, e92–e123. doi: 10.1111/j.1755-5949.2010.00154.x, 20557568 PMC6493805

[ref6] Bayram A. Yilmaz E. Z. (2024). The mediating role of spiral of silence in the effect of nomophobia on social withdrawal. Addicta 11, 117–124. doi: 10.5152/ADDICTA.2024.2024023

[ref7] Belmer A. Patkar O. L. Pitman K. M. Bartlett S. E. (2015). Serotonergic neuroplasticity in alcohol addiction. Brain. Plast. 1, 177–206. doi: 10.3233/BPL-150022, 29765841 PMC5928559

[ref8] Berridge C. W. (2008). Noradrenergic modulation of arousal. Brain Res. Rev. 58, 1–17. doi: 10.1016/j.brainresrev.2007.10.013, 18199483 PMC2517224

[ref9] Bertolín-Guillén J. M. (2023). The subjective need for social interaction. Br. J. Healthc. Med. Res. 10, 519–530.

[ref10] Boyce W. T. (2016). Differential susceptibility of the developing brain to contextual adversity and stress. Neuropsychopharmacology 41, 142–162. doi: 10.1038/npp.2015.294, 26391599 PMC4677150

[ref11] Braga-Dias A. C. Cardenas Otero S. J. Cavalcante Oliveira M. K. Canto-de-Souza L. Crestani C. C. Canto-de-Souza A. . (2025). Social isolation induces sex-differentiated sensory-emotional pain responses and oxytocinergic receptors alterations in mice. Eur. J. Pain 29:e70106. doi: 10.1002/ejp.70106, 40824064

[ref12] Brand M. Young K. S. Laier C. Wölfling K. Potenza M. N. (2016). Integrating psychological and neurobiological considerations regarding the development and maintenance of specific internet-use disorders: an interaction of person-affect-cognition-execution (I-PACE) model. Neurosci. Biobehav. Rev. 71, 252–266. doi: 10.1016/j.neubiorev.2016.08.033 27590829

[ref13] Brannan S. Garbe L. Richardson B. D. (2024). Early life stress induced sex-specific changes in behavior is paralleled by altered locus coeruleus physiology in BALB/cJ mice. Neurobiol. Stress 33:100674. doi: 10.1016/j.ynstr.2024.100674, 39385751 PMC11462065

[ref14] Cacioppo J. T. Hawkley L. C. Norman G. J. Berntson G. G. (2011). Social isolation. Ann. N. Y. Acad. Sci. 1231, 17–22. doi: 10.1111/j.1749-6632.2011.06028.x, 21651565 PMC3166409

[ref15] Campagne D. M. (2019). Stress and perceived social isolation (loneliness). Arch. Gerontol. Geriat. 82, 192–199. doi: 10.1016/j.archger.2019.02.007, 30825769

[ref16] Carcassi F. Sbardolini G. (2023). Assertion, denial, and the evolution of Boolean operators. Mind Lang. 38, 1187–1207. doi: 10.1111/mila.12448

[ref17] Chandavat A. S. Patel J. Bashir F. Chandavat A. S. (2025). Evaluation of dopamine and serotonin alterations in smartphone addiction and their impact on executive function. Int. J. Life. Sci. Biotechno. Pharm. Res. 14, 347–351.

[ref18] Cheng J. Y. Zhang S. W. Tai F. D. (2016). Effects of nucleus accumbens oxytocin and its antagonist on social approach behavior. Behav. Pharmacol. 27, 672–680. doi: 10.1097/FBP.0000000000000212, 26960161

[ref19] Cosme G. Patrocínio M. Cogoni C. Kosilo M. Prata D. (2025). Intranasal oxytocin increases cooperation of heterosexual men with women. Psychoneuroendocrinology 179:107519. doi: 10.1016/j.psyneuen.2025.107519, 40578004

[ref20] Covey D. P. Mateo Y. Sulzer D. Cheer J. F. Lovinger D. M. (2017). Endocannabinoid modulation of dopamine neurotransmission. Neuropharmacology 124, 52–61. doi: 10.1016/j.neuropharm.2017.04.033, 28450060 PMC5608040

[ref21] Cruces J. Venero C. Pereda-Peeez I. De la Fuente M. (2014). The effect of psychological stress and social isolation on neuroimmunoendocrine communication. Curr. Pharm. Des. 20, 4608–4628. doi: 10.2174/1381612820666140130205822, 24588822

[ref22] Cui C. Luo G. Lei J. Shi Y. Yao Y. Ren K. . (2025). Cannabinoid CB1 receptor in dopaminergic circuit from ventral tegmental area to nucleus accumbens links trait anxiety with reward learning. Transl. Psychiatry 15:395. doi: 10.1038/s41398-025-03644-5, 41073394 PMC12514215

[ref23] Dayan P. Balleine B. W. (2002). Reward, motivation, and reinforcement learning. Neuron 36, 285–298. doi: 10.1016/s0896-6273(02)00963-7, 12383782

[ref24] Dölen G. (2015). Autism: oxytocin, serotonin, and social reward. Soc. Neurosci. 10, 450–465. doi: 10.1080/17470919.2015.1087875, 26317636

[ref25] Dölen G. Malenka R. C. (2014). The emerging role of nucleus accumbens oxytocin in social cognition. Biol. Psychiatry 76, 354–355. doi: 10.1016/j.biopsych.2014.06.009, 25103539

[ref26] Duerler P. Vollenweider F. X. Preller K. H. (2022). A neurobiological perspective on social influence: serotonin and social adaptation. J. Neurochem. 162, 60–79. doi: 10.1111/jnc.15607, 35274296 PMC9322456

[ref27] Dunne G. (2025). Resisting edutainment: how reward deficiency syndrome fuels the student attention crisis. Stud. Philos. Educ. 45, 147–173. doi: 10.1007/s11217-025-10022-7

[ref28] Elhai J. D. Levine J. C. Dvorak R. D. Hall B. J. (2016). Fear of missing out, need for touch, anxiety and depression are related to problematic smartphone use. Comput. Hum. Behav. 63, 509–516. doi: 10.1016/j.chb.2016.05.079

[ref29] Fischer A. G. Ullsperger M. (2017). An update on the role of serotonin and its interplay with dopamine for reward. Front. Hum. Neurosci. 11:484. doi: 10.3389/fnhum.2017.00484, 29075184 PMC5641585

[ref30] Gabay S. Pertzov Y. Henik A. (2011). Orienting of attention, pupil size, and the norepinephrine system. Atten. Percept. Psychophys. 73, 123–129. doi: 10.3758/s13414-010-0015-4, 21258914

[ref31] Hallock H. L. Adiraju S. S. Miranda-Barrientos J. McInerney J. M. Oh S. DeBrosse A. C. . (2024). Electrophysiological correlates of attention in the locus coeruleus–prelimbic cortex circuit during the rodent continuous performance test. Neuropsychopharmacology 49, 521–531. doi: 10.1038/s41386-023-01692-3, 37563281 PMC10789747

[ref32] Haug S. Castro R. P. Kwon M. Filler A. Kowatsch T. Schaub M. P. (2015). Smartphone use and smartphone addiction among young people in Switzerland. J. Behav. Addict. 4, 299–307. doi: 10.1556/2006.4.2015.037, 26690625 PMC4712764

[ref33] Hazani R. Breton J. M. Trachtenberg E. Ruzal K. Shvalbo B. Kantor B. . (2025). Neural and behavioral correlates of individual variability in rat helping behavior: a role for social affiliation and oxytocin receptors. J. Neurosci. 45:e0845242025. doi: 10.1523/JNEUROSCI.0845-24.2025, 40295107 PMC12121707

[ref34] He H. Wang J. (2025). The roles of loneliness and self-control in the association between social avoidance and nomophobia among college students. Sci. Rep. 15:26782. doi: 10.1038/s41598-025-10789-2, 40702087 PMC12287310

[ref35] Hellriegel E. T. D'mello A. P. (1997). The effect of acute, chronic and chronic intermittent stress on the central noradrenergic system. Pharmacol. Biochem. Behav. 57, 207–214. doi: 10.1016/S0091-3057(96)00341-3 9164574

[ref36] Honma M. Masaoka Y. Iizuka N. Wada S. Kamimura S. Yoshikawa A. . (2022). Reading on a smartphone affects sigh generation, brain activity, and comprehension. Sci. Rep. 12:1589. doi: 10.1038/s41598-022-05605-0, 35102254 PMC8803971

[ref37] Horwood S. Anglim J. (2019). Problematic smartphone usage and subjective and psychological well-being. Comput. Hum. Behav. 97, 44–50. doi: 10.1016/j.chb.2019.02.028

[ref38] Hutchins N. Allen A. Curran M. Kannis-Dymand L. (2021). Social anxiety and online social interaction. Aust. Psychol. 56, 142–153. doi: 10.1080/00050067.2021.1890977

[ref39] Jahrami H. A. Fekih-Romdhane F. Saif Z. Q. Alhaj O. A. AlRasheed M. M. Pandi-Perumal S. R. . (2022). Sleep dissatisfaction is a potential marker for nomophobia in adults. Sleep Med. 98, 152–157. doi: 10.1016/j.sleep.2022.07.001, 35868112

[ref40] Jing M. Y. Ding X. Y. Han X. Zhao T. Y. Luo M. M. Wu N. . (2022). Activation of mesocorticolimbic dopamine projections initiates cue-induced reinstatement of reward seeking in mice. Acta Pharmacol. Sin. 43, 2276–2288. doi: 10.1038/s41401-022-00866-x, 35217811 PMC9433452

[ref41] Kanen J. W. Arntz F. E. Yellowlees R. Cardinal R. N. Price A. Christmas D. M. . (2021). Serotonin depletion amplifies distinct human social emotions as a function of individual differences in personality. Transl. Psychiatry 11:81. doi: 10.1038/s41398-020-00880-9, 33518708 PMC7847998

[ref42] Karayol R. Medrihan L. Warner-Schmidt J. L. Fait B. W. Rao M. N. Holzner E. B. . (2021). Serotonin receptor 4 in the hippocampus modulates mood and anxiety. Mol. Psychiatry 26, 2334–2349. doi: 10.1038/s41380-020-00994-y, 33441982 PMC8275670

[ref43] Kardefelt-Winther D. (2014). A conceptual and methodological critique of internet addiction research: towards a model of compensatory internet use. Comput. Human. Behav. 31, 351–354. doi: 10.1016/j.chb.2013.10.059

[ref44] Koch S. B. van Zuiden M. Nawijn L. Frijling J. L. Veltman D. J. Olff M. (2016). Intranasal oxytocin normalizes amygdala functional connectivity in posttraumatic stress disorder. Neuropsychopharmacology 41, 2041–2051. doi: 10.1038/npp.2016.1, 26741286 PMC4908648

[ref45] Kranz G. S. Kasper S. Lanzenberger R. (2010). Reward and the serotonergic system. Neuroscience 166, 1023–1035. doi: 10.1016/j.neuroscience.2010.01.036, 20109531

[ref46] Kross E. Verduyn P. Demiralp E. Park J. Lee D. S. Lin N. . (2013). Facebook use predicts declines in subjective well-being in young adults. PLoS One 8:e69841. doi: 10.1371/journal.pone.0069841, 23967061 PMC3743827

[ref47] Levine L. E. Waite B. M. Bowman L. L. (2012). Mobile media use, multitasking and distractibility. Int. J. Cyber Behav. Psychol. Learn. 2, 15–29. doi: 10.4018/ijcbpl.2012070102

[ref48] Li T. M. Wong P. W. (2015). Youth social withdrawal behavior (hikikomori): a systematic review of qualitative and quantitative studies. Aust. N. Z. J. Psychiatry 49, 595–609. doi: 10.1177/0004867415581179, 25861794

[ref49] Ma S. T. Abelson J. L. Okada G. Taylor S. F. Liberzon I. (2017). Neural circuitry of emotion regulation: effects of appraisal, attention, and cortisol administration. Cogn. Affect. Behav. Neurosci. 17, 437–451. doi: 10.3758/s13415-016-0489-1, 28032303

[ref50] Ma X. Zhao W. Luo R. Zhou F. Geng Y. Xu L. . (2018). Sex-and context-dependent effects of oxytocin on social sharing. NeuroImage 183, 62–72. doi: 10.1016/j.neuroimage.2018.08.004, 30086408

[ref51] Macit H. B. Macit G. Güngör O. (2018). A research on social media addiction and dopamine driven feedback. Mehmet Akif Ersoy Üniversitesi İktisadi ve İdari Bilimler Fakültesi Dergisi 5, 882–897. doi: 10.30798/makuiibf.435845

[ref52] Martinsson P. Thomée S. (2025). Co-worker phubbing: a qualitative exploration of smartphone use during work breaks. Scand. J. Psychol. 66, 158–173. doi: 10.1111/sjop.13071, 39300692 PMC11735245

[ref53] Marty-Dugas J. Ralph B. C. Oakman J. M. Smilek D. (2018). The relation between smartphone use and everyday inattention. Psychol. Conscious. Theory Res. Pract. 5, 46–62. doi: 10.1037/cns0000131

[ref54] McDaniel B. T. Radesky J. S. (2018). Technoference: longitudinal associations between parent technology use, parenting stress, and child behavior problems. Pediatr. Res. 84, 210–218. doi: 10.1038/s41390-018-0052-6, 29895837 PMC6185759

[ref55] Meinhardt L. M. Elhaidary M. Colley M. Rietzler M. Rixen J. O. Purohit A. K. . (2025). Scrolling in the deep: analysing contextual influences on intervention effectiveness during infinite scrolling on social media. Proceedings of the 2025 CHI Conference on Human Factors in Computing Systems, 1–17.

[ref56] Menon R. Neumann I. D. (2023). Detection, processing and reinforcement of social cues: regulation by the oxytocin system. Nat. Rev. Neurosci. 24, 761–777. doi: 10.1038/s41583-023-00759-w, 37891399

[ref57] Mert İ. S. Şen C. Abubakar A. M. (2025). Impact of social media usage on technostress and cyber incivility. Inform. Dev. 41, 1407–1422. doi: 10.1177/02666669231204954

[ref58] Misra S. Cheng L. Genevie J. Yuan M. (2016). The iPhone effect: the quality of in-person social interactions in the presence of mobile devices. Environ. Behav. 48, 275–298. doi: 10.1177/0013916514539755

[ref59] Mohamed S. A. A. K. Shaban M. (2025). Digital dependence in aging: nomophobia as the new mental health threat for older adults. J. Emerg. Nurs. 51, 371–378. doi: 10.1016/j.jen.2025.02.001, 40340052

[ref60] Mumtaz F. Khan M. I. Zubair M. Dehpour A. R. (2018). Neurobiology and consequences of social isolation stress in animal model-a comprehensive review. Biomed. Pharmacother. 105, 1205–1222. doi: 10.1016/j.biopha.2018.05.086, 30021357

[ref61] Nishi M. (2020). Effects of early-life stress on the brain and behaviors: implications of early maternal separation in rodents. Int. J. Mol. Sci. 21:7212. doi: 10.3390/ijms21197212, 33003605 PMC7584021

[ref62] Ohline S. M. Abraham W. C. (2019). Environmental enrichment effects on synaptic and cellular physiology of hippocampal neurons. Neuropharmacology 145, 3–12. doi: 10.1016/j.neuropharm.2018.04.007, 29634984

[ref63] Olff M. Frijling J. L. Kubzansky L. D. Bradley B. Ellenbogen M. A. Cardoso C. . (2013). The role of oxytocin in social bonding, stress regulation and mental health: an update on the moderating effects of context and interindividual differences. Psychoneuroendocrinology 38, 1883–1894. doi: 10.1016/j.psyneuen.2013.06.019, 23856187

[ref64] Ott T. Nieder A. (2019). Dopamine and cognitive control in prefrontal cortex. Trends Cogn. Sci. 23, 213–234. doi: 10.1016/j.tics.2018.12.006, 30711326

[ref65] Oyasor E. I. (2025). Cognitive load and smartphones: how mere presence impacts mental performance. Pak. J. Life. Soc. Sci. 23, 760–772. doi: 10.57239/PJLSS-2025-23.2.00208

[ref66] Papasteri C. C. Sofonea A. Boldasu R. Poalelungi C. Tomescu M. I. Pistol C. A. . (2020). Social feedback during sensorimotor synchronization changes salivary oxytocin and behavioral states. Front. Psychol. 11:531046. doi: 10.3389/fpsyg.2020.531046, 33071856 PMC7538614

[ref67] Parasuraman S. (2023). Nomophobia may increase the risk of anxiety, depression, and social isolation. Free. Radic. Antioxid. 13, 1–2. doi: 10.5530/fra.2023.1.1

[ref68] Patulny R. Seaman C. (2017). ‘I’ll just text you’: is face-to-face social contact declining in a mediated world? J. Sociol. 53, 285–302. doi: 10.1177/1440783316674358

[ref69] Pazdur M. Tutus D. Haag A. C. (2025). Risk factors for problematic social media use in youth: a systematic review of longitudinal studies. Adolesc. Res. Rev. 10, 237–253. doi: 10.1007/s40894-025-00264-4, 30311153

[ref70] Pietrabissa G. Simpson S. G. (2020). Psychological consequences of social isolation during COVID-19 outbreak. Front. Psychol. 11:2201. doi: 10.3389/fpsyg.2020.02201, 33013572 PMC7513674

[ref71] Roberts J. A. David M. E. (2023). On the outside looking in: social media intensity, social connection, and user well-being: the moderating role of passive social media use. Can. J. Behav. Sci. Rev. Can. Sci. Comport. 55, 240–252. doi: 10.1037/cbs0000323

[ref72] Roffarello A. M. De Russis L. (2021). Understanding, discovering, and mitigating habitual smartphone use in young adults. ACM Trans. Interact. Intell. Syst. 11, 1–34. doi: 10.1145/3447991

[ref73] Runions K. C. Morandini H. A. Rao P. Wong J. W. Kolla N. J. Pace G. . (2019). Serotonin and aggressive behaviour in children and adolescents: a systematic review. Acta Psychiatr. Scand. 139, 117–144. doi: 10.1111/acps.12986 30446991

[ref74] Russell G. Lightman S. (2019). The human stress response. Nat. Rev. Endocrinol. 15, 525–534. doi: 10.1038/s41574-019-0228-0, 31249398

[ref75] Saxbe D. E. (2008). A field (researcher's) guide to cortisol: tracking HPA axis functioning in everyday life. Health Psychol. Rev. 2, 163–190. doi: 10.1080/17437190802530812

[ref76] Sbarra D. A. Briskin J. L. Slatcher R. B. (2019). Smartphones and close relationships: the case for an evolutionary mismatch. Perspect. Psychol. Sci. 14, 596–618. doi: 10.1177/1745691619826535, 31002764

[ref77] Schwarz L. A. Luo L. (2015). Organization of the locus coeruleus-norepinephrine system. Curr. Biol. 25, R1051–R1056. doi: 10.1016/j.cub.2015.09.039, 26528750

[ref78] Sherman L. E. Hernandez L. M. Greenfield P. M. Dapretto M. (2018). What the brain ‘likes’: neural correlates of providing feedback on social media. Soc. Cogn. Affect. Neurosci. 13, 699–707. doi: 10.1093/scan/nsy051, 29982823 PMC6121147

[ref79] Silva H. M. (2025). The reconfiguration of social bonds in the digital age: virtual connections vs. face-to-face relationships. Nat. Anthropol. 3, 10003–10003. doi: 10.70322/natanthropol.2025.10003

[ref80] Son Y. Oh W. (2023). Digitalization of loyalty: impacts of mobile technology on reward redemption and engagement level. J. Manag. Inf. Syst. 40, 1301–1327. doi: 10.1080/07421222.2023.2267313

[ref81] Srivastava S. Verma N. Kumar D. Singh N. Kumar K. (2025). Nomophobia as an emerging psychopathology psychophysiological mechanisms and clinical implications. Ann. Neurosci. 09727531251351082. doi: 10.1177/09727531251351082, 40686540 PMC12274209

[ref82] Sustkova-Fiserova M. Puskina N. Havlickova T. Lapka M. Syslova K. Pohorala V. . (2020). Ghrelin receptor antagonism of fentanyl-induced conditioned place preference, intravenous self-administration, and dopamine release in the nucleus accumbens in rats. Addict. Biol. 25:e12845. doi: 10.1111/adb.12845, 31696597

[ref83] Takemoto T. Baba M. Yokoyama K. Kitagawa K. Nagayasu K. Ago Y. . (2022). Oxytocin ameliorates impaired social behavior in a mouse model of 3q29 deletion syndrome. Mol. Brain 15:26. doi: 10.1186/s13041-022-00915-w, 35346312 PMC8962454

[ref84] Tanaka S. C. Samejima K. Okada G. Ueda K. Okamoto Y. Yamawaki S. . (2006). Brain mechanism of reward prediction under predictable and unpredictable environmental dynamics. Neural Netw. 19, 1233–1241. doi: 10.1016/j.neunet.2006.05.039, 16979871

[ref85] Thangavel V. (2024). Nomophobia in India: a psychological disorder that causes the brain to release dopamine in response to tweets, emoticons, and other acts, rewarding the behavior and sustaining the habit of using social media addiction. Curr. Trends Mass. Commun. 3, 01–16. doi: 10.2139/ssrn.4636001

[ref86] Thomson E. M. (2019). Air pollution, stress, and allostatic load: linking systemic and central nervous system impacts. J. Alzheimer's Dis 69, 597–614. doi: 10.3233/JAD-190015, 31127781 PMC6598002

[ref87] Tian Z. Yamanaka M. Bernabucci M. Zhao M. G. Zhuo M. (2017). Characterization of serotonin-induced inhibition of excitatory synaptic transmission in the anterior cingulate cortex. Mol. Brain 10:21. doi: 10.1186/s13041-017-0303-1, 28606116 PMC5468981

[ref88] Tufan C. Köksal K. Griffiths M. D. Erturgut R. Mert İ. S. (2025). Effects of fear of missing out, smartphone addiction, phubbing, and being phubbed on friendship satisfaction. Behav. Inf. Technol. 44, 3592–3608. doi: 10.1080/0144929X.2024.2434893

[ref89] Uhls Y. T. Michikyan M. Morris J. Garcia D. Small G. W. Zgourou E. . (2014). Five days at outdoor education camp without screens improves preteen skills with nonverbal emotion cues. Comput. Hum. Behav. 39, 387–392. doi: 10.1016/j.chb.2014.05.036

[ref90] van Stegeren A. H. Wolf O. T. Everaerd W. Scheltens P. Barkhof F. Rombouts S. A. (2007). Endogenous cortisol level interacts with noradrenergic activation in the human amygdala. Neurobiol. Learn. Mem. 87, 57–66. doi: 10.1016/j.nlm.2006.05.008, 16884932

[ref91] Verdejo-Garcia A. Moreno-Padilla M. Garcia-Rios M. C. Lopez-Torrecillas F. Delgado-Rico E. Schmidt-Rio-Valle J. . (2015). Social stress increases cortisol and hampers attention in adolescents with excess weight. PLoS One 10:e0123565. doi: 10.1371/journal.pone.0123565, 25898204 PMC4405363

[ref92] Verduyn P. Schulte-Strathaus J. C. Kross E. Hülsheger U. R. (2021). When do smartphones displace face-to-face interactions and what to do about it? Comput. Hum. Behav. 114:106550. doi: 10.1016/j.chb.2020.106550

[ref93] Vetulani J. (2013). Early maternal separation: a rodent model of depression and a prevailing human condition. Pharmacol. Rep. 65, 1451–1461. doi: 10.1016/s1734-1140(13)71505-6, 24552992

[ref94] Vogels E. A. Gelles-Watnick R. Massarat N. (2022). Teens, social media and technology 2022. Pew Res. Center 10, 1–30.

[ref95] Vogt B. Bengart P. Declerck C. Fehr E. (2025). Oxytocin increases trust in humans with a low disposition to trust. bioRxiv:2025-2110. doi: 10.1101/2025.10.01.679711

[ref96] Volkow N. D. Wang G. J. Fowler J. S. Tomasi D. Telang F. Baler R. (2010). Addiction: decreased reward sensitivity and increased expectation sensitivity conspire to overwhelm the brain's control circuit. BioEssays 32, 748–755. doi: 10.1002/bies.201000042, 20730946 PMC2948245

[ref97] Wajahat A. (2024). Social media and its impact on non-verbal communication. IJFMR. 6, 1–10. doi: 10.36948/ijfmr.2024.v06i03.23102

[ref98] Walsh M. J. Clark S. J. (2019). Co-present conversation as “socialized trance”: talk, involvement obligations, and smart-phone disruption. Symb. Interact. 42, 6–26. doi: 10.1002/symb.382

[ref99] Wang C. Lee M. K. (2020). Why we cannot resist our smartphones: investigating compulsive use of mobile SNS from a stimulus-response-reinforcement perspective. J. Assoc. Inf. Syst. 21, 175–200. doi: 10.17705/1jais.00596

[ref100] Wang Z. Yang X. Zhang X. (2020). Relationships among boredom proneness, sensation seeking and smartphone addiction among Chinese college students: mediating roles of pastime, flow experience and self-regulation. Technol. Soc. 62:101319. doi: 10.1016/j.techsoc.2020.101319

[ref101] Warren C. M. Wilson R. C. van der Wee N. J. Giltay E. J. van Noorden M. S. Bosch J. A. . (2017). The effect of atomoxetine on directed and random exploration in humans. PLoS One 12, 1–17. doi: 10.1371/journal.pone.0176034 PMC5405969 28445519

[ref102] Wenzel J. M. Cheer J. F. (2018). Endocannabinoid regulation of reward and reinforcement through interaction with dopamine and endogenous opioid signaling. Neuropsychopharmacology 43, 103–115. doi: 10.1038/npp.2017.126, 28653666 PMC5719091

[ref103] Yao S. Kendrick K. M. (2025). How does oxytocin modulate human behavior? Mol. Psychiatry 30, 1639–1651. doi: 10.1038/s41380-025-02898-1, 39827220

[ref104] Yeğen B. (2010). Oxytocin and hypothalamo-pituitaryadrenal axis. Marmara Pharm. J. 14, 61–66. doi: 10.12991/201014450

[ref105] Yu W. Yen Y. C. Lee Y. H. Tan S. Xiao Y. Lokman H. . (2019). Prenatal selective serotonin reuptake inhibitor (SSRI) exposure induces working memory and social recognition deficits by disrupting inhibitory synaptic networks in male mice. Mol. Brain 12:29. doi: 10.1186/s13041-019-0452-5, 30935412 PMC6444596

[ref106] Zavaleta D. Samuel K. Mills C. T. (2017). Measures of social isolation. Soc. Indic. Res. 131, 367–391. doi: 10.1007/s11205-016-1252-2

[ref107] Zhao W. Geng Y. Luo L. Zhao Z. Ma X. Xu L. . (2017). Oxytocin increases the perceived value of both self-and other-owned items and alters medial prefrontal cortex activity in an endowment task. Front. Hum. Neurosci. 11:272. doi: 10.3389/fnhum.2017.00272, 28588469 PMC5440465

[ref108] Zona L. C. Fry B. R. LaLonde J. A. Cromwell H. C. (2017). Effects of anandamide administration on components of reward processing during free choice. Pharmacol. Biochem. Behav. 158, 14–21. doi: 10.1016/j.pbb.2017.05.008, 28529018

